# Treatment of Primary Axillary Hyperhidrosis with Two Doses of Botulinum Toxin A—Observational Study

**DOI:** 10.3390/toxins16070320

**Published:** 2024-07-16

**Authors:** María Jesús Antón Andrés, Ernesto Domingo Candau Pérez, María Pilar Bermejo de la Fuente

**Affiliations:** Physical Medicine and Rehabilitation Service, Río Hortega University Hospital, 47012 Valladolid, Spain; ecandaup@saludcastillayleon.es (E.D.C.P.); pbermejof@saludcastillayleon.es (M.P.B.d.l.F.)

**Keywords:** axillary hyperhidrosis, botulinum toxin, onabotulinum toxin, quality of life, hyperhidrosis severity scale, dermatology quality of life index

## Abstract

Hyperhidrosis (HH) is defined as the production of more sweat than is necessary for its thermoregulatory function, negatively affecting patients’ quality of life and interfering with their social, work and family life. In this context, the aim of thisstudy was to evaluate the efficacy of two different doses of botulinum toxin type A (50 or 100 units) in each axilla in severe primary axillary hyperhidrosis. A descriptive, observational, cross-sectional and post-authorisation study was conducted onpatients referred to our department.Thirty-one patients with severe primary axillary hyperhidrosis were included, some of whom received more than one infiltration during the follow-up period, performing a total of 82 procedures. They were assigned by simple random sampling to two types of treatment: infiltration of 50 or 100 units (U) of botulinum toxin A per axilla.Hyperhidrosis severity was assessed using the Hyperhidrosis Disease Severity Scale (HDSS), and quality of life was assessed using the Dermatology Life Quality Index (DLQI) questionnaire. Onabotulinum toxin A infiltration reduced the severity of hyperhidrosis and improved the quality of life of the treated patients, with no significant differences between the two groups.

## 1. Introduction

HH is defined as the production of more sweat than is necessary for its thermoregulatory function, being perceptible, unpredictable and involuntary. It is a common but underdiagnosed condition, which causes an alteration in body aesthetics, negatively affecting the quality of life of patients who suffer from it and interfering with their social, work and family life [1].

Although excessive sweating can appear at any age, palmar and plantar hyperhidrosis usually begins in childhood, axillary hyperhidrosis in adolescence due to enlargement of the apocrine glands and craniofacial hyperhidrosis in adulthood, with a frequent family history when it appears before the age of 20 [2].

There is a wide variation in the prevalence of primary HH worldwide, from as low as 2.8% to as high as 10% [3,4,5].

It is classified according to distribution as focal (mainly affecting palms, soles, axillae and craniofacial region) or generalised, according to aetiology as primary or secondary, and according to severity as mild, moderate or severe [6].

Approximately 93% of patients have primary HH, usually localised, bilateral and symmetrical, with no nocturnal onset. The cause is unknown, although it may be due to a complex dysfunction of the autonomic nervous system, resulting in hyperactivity of the sympathetic system that innervates the sweat glands [6].

Secondary HH is usually generalised and asymmetrical, although there may be cases with focal presentation, such as Frey’s syndrome or auriculotemporal syndrome [7,8], or stump hyperhidrosis [9]. When it is generalised, it may be due to infections, neurological, endocrine, tumour or drug-related disorders, among others [2,10].

Diagnosis is based on anamnesis and clinical findings, and there is no test that helps to confirm or rule it out. A main criterion for diagnosis is focal, visible and excessive sweating of at least six months duration without apparent cause, with at least two secondary criteria: bilateral and relatively symmetrical, impact on the activities of daily living, at least one episode per week, onset before the age of 25 years and a family history of idiopathic hyperhidrosis [11].

Topical antiperspirants with aluminium salts are recommended as the first treatment option in mild hyperhidrosis, and topical anticholinergics such as glycopyrronium can also be used [12]. Iontophoresis has been shown to be effective in the short term on the palms and soles, but not in the axillae due to the difficulty of its application given the characteristic anatomy of the area. In case of non-response, botulinum toxin type A, onabotulinum toxin, approved by the FDA in 2004 [13] for severe primary axillary hyperhidrosis, is used as a second-choice treatment.

A consensus has not been well established on toxin infiltration protocols, neither in terms of dilution nor in terms of the number of points to infiltrate per axilla or the recommended doses [14,15,16,17,18,19,20], which is why we considered it interesting to carry out this study to try to provide information on the most appropriate dose for controlling symptoms for as long as possible, improving quality of life and body aesthetics and without increasing side effects in the patient.

The main objective of the study was to assess the efficacy using the Hyperhidrosis Disease Severity Scale (HDSS), the quality of life using the Dermatology Life Quality Index (DLQI) and the safety of two different doses (50 U vs. 100 U in each axilla) of botulinum toxin type A, in severe primary axillary hyperhidrosis, in real clinical practice. At the end of the study, the PGI-I (Patient Global Impression of Improvement) scale was administered.

## 2. Results

Thirty-one patients aged between 15 and 52 years with a diagnosis of severe primary axillary hyperhidrosis were includedand divided into two groups according to the dose of toxinreceived. The mean age of the patients was 33 years, with a predominance of the female sex, and there were no significant differences between the two groups. In the rest of the variables studied, no significant differences were found between groups (Table 1).

Throughout the study, some patients required more than one infiltration as the toxin wore off and hyperhidrosis returned, and a total of 82 procedures were performed.

The number of procedures performed was similar in both groups, 40 in the 50 U toxin group in each axilla and 42 in the 100 U group. At baseline, the mean HDSS scale showed no significant difference between the two groups, and the DLQI scale was approximately two points lower in the 100 U per axilla group, but with no significant difference between groups (Table 2).

The highest scores on the DLQI scale (response much or very much) were recorded for questions related to feeling embarrassed or self-conscious, interference with social activities and stains on clothes. The table shows the data collected and the degree of significance (Table 3).

**Figure 1:**
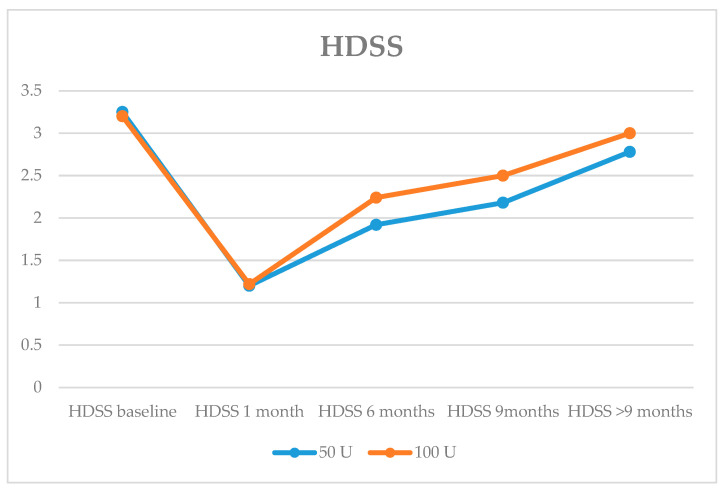
Evolution of mean HDSS (Hyperhidrosis Disease Severity Scale) at baseline and at check-ups at 1 month, 6 months, 9 months and >9 months.

On the HDSS scale, a decrease in the mean of 2 points at one month of treatment was observed in both groups, with a decrease of more than one point persisting at the 9-month follow-up in the 50 U group and 0.7 points in the 100 U group, with no significant differences (Figure 1).

**Figure 2:**
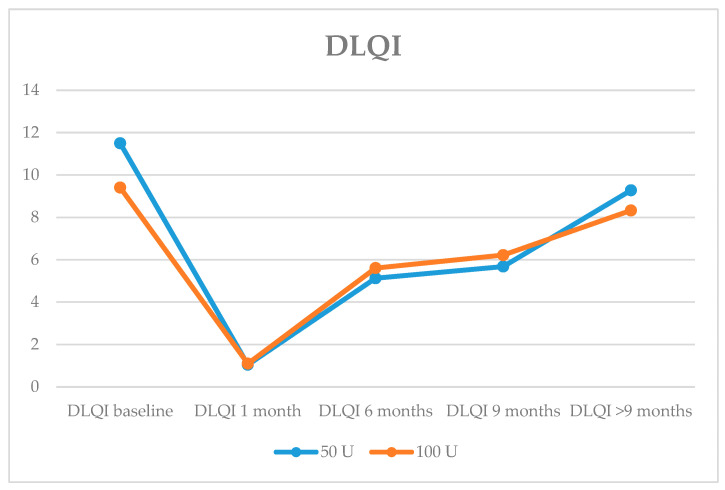
Evolution of mean DLQI (Dermatology Life Quality Index) scale at baseline and at check-ups at 1 month, 6 months, 9 months and >9 months.

On the DLQI scale, the mean decrease was more than 10 points after one month of treatment in the 50 U group, and more than 9 points in the 100 U group. At 9 months after treatment, the improvement persisted, with a mean of 5.68 in the 50 U group and 6.22 in the 100 U group. No significant differences were found between the two groups (Figure 2).

To assess treatment outcomes, four groups or pairs were established for each of the scales. Pair 1 showed the baseline results and the results one month after treatment. Pair 2 recorded the patients’ baseline data and six months after treatment. Pair 3 showed baseline data and data at 9 months of treatment, and finally Pair 4, which reflected baseline data and data from patients who continued to report sweating control, score 1 or 2 on the HDSS scale, beyond 9 months of infiltration.

Table 4 shows the differences between the baseline mean and the mean at successive revisions in both the HDSS and DLQI scales in both treatment groups, as well as the number of patients who remained in control of sweating (HDSS 1 or 2) at each of the revisions, with no significant differences between the two groups.

In the 50 U group, one patient required a new infiltration before 6 months and 12 patients before 9 months, with 18 patients in whom the improvement in sweating was greater than 9 months, and no repeat treatment was indicated at that time. In the 100U group, none of the 42 procedures performed required re-infiltration before 6 months, 18 required re-infiltration before 9 months, and in 12 patients, the improvement was sustained beyond 9 months.

At the end of each infiltration, the Patient Global Impression of Improvement scale, PGI-I, was administered. In both groups, practically all patients reported feeling “much better” or “better” than before treatment. There were no patients with a negative result, and the difference between the two groups was not significant (Table 5).

## 3. Discussion

Primary hyperhidrosis is a common condition that has a negative effect on the quality of life of patients, interfering with their social, work and family life. It has been accepted that its frequency is underestimated, either because patients do not consult this problem or because it is not diagnosed by health professionals. Doolittle in 2016 describes that only 51% of patients with hyperhidrosis had consulted their doctor, and of these, only 53% were diagnosed with hyperhidrosis [3]. Glaser et al. report that 85% of patients with hyperhidrosis waited more than 3 years before consulting a physician, and almost half of them more than 10 years [21].

In our series, we found that 51.6% of patients had a family history of hyperhidrosis, which is a result in line with other authors, such as Henning et al., with percentages between 35–56% [22]. Quality of life is impaired in up to 80% of patients with HH, with a higher likelihood of developing anxiety or depression than in patients without HH [23,24].

The development of the DLQI in 1994 quickly contributed to its use in clinical trials to assess improvement in quality of life in parallel with clinical severity measures for different dermatological conditions [25], helping to measure quantitatively what previously could only be explained by subjective patient feedback.

The use of botulinum toxin for hyperhidrosis is well documented in the medical literature, being the treatment of choice in patients with severe primary axillary hyperhidrosis who have not responded to topical treatment with aluminium salts. In Spain, onabotulinum toxin A is only approved for axillary hyperhidrosis, and there is no commercial authorisation for its use in other locations, although there are many published studies of its use in clinical practice with good results.

In our study, the mean baseline HDSS scores in both groups were 3.25 ± 0.44 and 3.19 ± 0.39, somewhat lower than those described in other series [26]. The mean pre-treatment DLQI scores were 11.5 ± 5.9 and 9.4 ± 3.1, respectively, similar to the results found by other authors of values between 10.1 and12.1, and lower than those described by Castiglione L [27]. These scores indicate a moderate to severe negative effect of hyperhidrosis on quality of life [28].

At one month after treatment, the mean decrease in the HDSS scale was 2.1 ± 0.6 in the 50 U group, and 1.9 ± 0.5 in the 100 U group, which represents a reduction of approximately 80%. At 6 months, a decrease of 1.3 ± 0.9 was maintained in 97.5% of patients in the first group and 0.9 ± 0.7 in 100% of patients in the 100 U toxin group. These results with significant improvement for at least 6 months are similar to those described by authors such as Naumann [29] or Stolman LP [30]; however, we did not find significant differences between the two groups, contrary to what is described by the latter who managed to extend the response with the infiltration of doses greater than 50 U per axilla.

Quality of life improved in both groups after treatment, with a decrease of 10 points in the 50 U group and 8 points in the 100 U group 1month after treatment, and 6 points and almost 4 points, respectively, at 6 months, in line with the results described by other authors such as Kouris A [31] and Mirkovic SE [32].

No significant side effects other than mild pain at the site of infiltration were reported [18]. None of the patients reported compensatory sweating either, although rates of around 4% have been reported, being reversible in case of occurrence.

A limitation of our study is the sample size. During the follow-up period we were only able to recruit 31 patients, perhaps due to the fact that despite being a very frequent entity, patients consult very little. All procedures performed during the follow-up period were included, with a total of 82, in order to improve the results.

## 4. Conclusions

Based on the results of our study, we can conclude that the infiltration of botulinum toxin type A is an effective, safe and minimally invasive therapy that reduces the severity of sweating and improves the quality of life of patients with severe primary axillary hyperhidrosis who have not responded to topical treatment, with no significant differences between the infiltration of 50 U or 100 U of onabotulinum toxin A per axilla. This study could contribute to unifyingthe dosage of onabotulinum toxin A for the conservative treatment of this common and disabling pathology.

## 5. Materials and Methods

An observational, descriptive, cross-sectional study was designed. Patients were recruited from the Physical Medicine and Rehabilitation Service of the Río Hortega University Hospital in Valladolid, Spain, referred from Primary Care or Dermatology with severe primary axillary hyperhidrosis. Patients were assigned to one of the two study groups by simple randomisation.

Inclusion criteria were patients over 14 years of age with severe primary axillary hyperhidrosis that did not improve with topical treatments. All patients signed the informed consent document for participation in the study and for toxin infiltration.

Exclusion criteria included a previous treatment with botulinum toxin within the last 3 months, a known allergy to botulinum toxin type A or the excipients of the formulation, fever or local infection at the infiltration site, pregnancy, lactation and motor neuron or neuromuscular transmission diseases.

The study was conducted in compliance with all applicable laws and regulations, in accordance with international ethical principles, primarily the Declaration of Helsinki and the ICH (International Conference of Harmonization) Standards of Good Clinical Epidemiological Practice. Patients were identified by a code, and their data were treated with absolute confidentiality. The study was approved by the Clinical Research Ethics Committee (CEIC) of the Valladolid West Health Area, with internal code CEIC 142/17.

For data collection, a database was created in Excel and then exported to the SPSS 15.0 statistical programme, which was used for statistical analysis.

For the descriptive statistical analysis, the normality of the quantitative variables was established with the Kolmogorov–Smirnov test, described as mean ± standard deviation. Paired comparisons were made for each of the procedures performed (basal vs. follow-up) using the paired *t*-test or Wilcoxon test. To study differences between independent means (50 U vs. 100U), the unpaired Student’s *t*-testor Mann–Whitney test was used. Fisher’s exact test was used to study the association between variables, which allows us to analyse whether two dichotomous variables are associated when the sample to be studied is small.A significance level of 5% was established.

The HDSS is the most commonly used scale to assess the severity of hyperhidrosis [16] and itsimpact on activities of daily living. It consists of 4 possible answers to the question *how would you rate the severity of your hyperhidrosis?* with 1 indicating ‘never noticeable’, 2 ‘tolerable’, 3 ‘barely tolerable’ and 4‘intolerable’. A score of 1 was indicative of mild hyperhidrosis and 2 indicated moderate hyperhidrosis whereas scores of 3 or 4 pointed to severe hyperhidrosis. A reduction of 1point on this scale is considered to correspond to a decrease in sweating of approximately 50%, and 2 points to a reduction of 80%. A decrease of 1 point post-treatment was defined as a good outcome.

The DLQI [33] is a quality-of-life questionnaire for use in dermatology published in 1994, with a time frame covering the last week. It consists of 10 items, each of which includes a Likert-type scale with four possible responses: very much, much, a little or not at all, with the scores being 3, 2, 1 and 0, respectively. A value of 3 indicates a worse quality of life. The total score is obtained by adding the score obtained in each of the items, ranging from 0 to 30 points. We have defined a decrease of 4 points as the minimum clinically important difference [34].

The PGI-I consists of a single question in which the patient is asked to rate the relief obtained from the treatment. It follows a seven-point Likert-type scale: very much better, much better, a little better, no change, a little worse, much worse or very much worse.

Botulinum toxin infiltration was performed in the rehabilitation department of our hospital. The hyperhidrosisareawas delimited by visual inspection in those patients with visible hyperhidrosis at the time of toxin infiltration and with the Minor’s Test in those who could not visually delimit the hyperhidrosis area.

The botulinum toxin type A used was onabotulinum toxin A (BOTOX, Allergan, Inc., Irvine, CA, USA), in the form of 100 U per vial, diluted in 2 mL of 0.9% saline solution. There is no consensus on dilution protocols for onabotulimtoxinA for the treatment of primary hyperhidrosis, and it can be prepared using 1 to 10 mL of physiological saline solution, although most clinicians use 2 to 5 mL [17]. The technical data sheet for onabotulinum toxin A (Botox) reports the reconstitution of the botulinum toxin with physiological saline but does not indicate the volume to be used, so the dilution was carried out following the method used by authors such as Glasser A [35].We used 1 mL syringes graduated in 10 units of 0.1 mL and sterile 30-gauge needles.

There is no consensus on the number of botulinum toxin infiltration sites in the axilla; recent authors have evaluated the effectiveness of toxin with fewer infiltration sites with good results [19]. In our study, the axilla was divided into ten squares and was made on the basis of previous studies, whichperformed10 to 15 infiltrations per axilla [29,35], each of which was infiltrated with 5 U (for the dose of 50 U per axilla) or 10 U (for the dose of 100 U per axilla) of toxin.

In the axilla, it is recommended that the toxin be administered by intradermal injections into the dermo-subcutaneous junction which can be found in this location at a depth of about 2 mm. The accuracy of infiltration is difficult, with studies reporting that they have found no difference in the efficacy of botulinum toxin type A in the treatment of axillary hyperhidrosis when administered by subcutaneous or intradermal injection [19,36]. The needle was inserted at an angle of approximately 45° to the surface of the dermis, a position also used by other authors [17], and at a depth of 2 mm.

To reduce the pain, in some patients, the injection wasperformedafter applying ice spray [37].

Patients were assessed prior to toxin infiltration, at 1 month, 6 months, 9 months and after 9 months.

## Figures and Tables

**Table 1:** Description of study variables.

	50 U TOXIN	100 U TOXIN	Significance
	N = 18	N = 13	
AGE	33.22 ± 10.55	33.62 ± 10.24	0.918
SEX			0.676
Male	3 (16.7%)	3 (23.1%)	
Female	15 (83.3%)	10 (76.9%)	
CLINIC START			0.965
<10 years	6 (33.3%)	4 (30.8%)	
10–15 years	8 (44.4%)	4 (30.8%)	
15–20 years	2 (11.1%)	3 (23.1%)	
>20 years	2 (11.1%)	2 (15.3%)	
FAMILY HISTORY			0.897
No	9 (50%)	6 (46.2%)	
Yes	9 (50%)	7 (53.8%)	
BILATERALITY			
Yes	18 (100%)	13 (100%)	0.999
PALMS			
Yes	8 (44.4%)	9 (69.2%)	0.171
PLANTS			
Yes	8 (44.4%)	9 (69.2%)	0.171
LOCATIONS OTHER THAN PALMS AND PLANTS			
Head and neck	1 (5.6%)	0 (0%)	0.868
Trunk	2 (11.1%)	0 (0%)	0.615
Others	1 (5.6%)	2 (15.4%)	0.765
ASSOCIATED SYMPTOMS			
Coldness	2 (11.1%)	5 (38.5%)	0.173
Itching	2 (11.1%)	2 (15.4%)	0.847
NIGHT SWEATS			
No	18 (100%)	13 (100%)	0.999
TRIGGERING FACTORS			
Heat	0 (0%)	0 (0%)	0.999
Stress	1 (5.6%)	1 (7.7%)	0.615
Heat and stress	13 (72.2%)	9 (69.2%)	0.826
Always/no stimulus	4 (22.2%)	3 (23.1%)	0.704

**Table 2:** Mean ofscales HDSS (Hyperhidrosis Disease Severity Scale) and DLQI (Dermatology Life Quality Index) in both groups.

	50 U N = 40	100 U N = 42	Significance
**HDSSMean**	3.25 ± 0.44	3.19 ± 0.39	0.521
**DLQI Mean**	11.50 ± 5.91	9.36 ± 3.11	0.280

**Table 3:** The scoresofeach of the questions of the DLQI (Dermatology Life Quality Index) scale in the 50 U and 100 U toxin per axilla groups. *p* (significance).

		DOSE		Total N%	*p*
		50 U N = 40	100 U N = 42		
		N%	N%		
**Itching, Discomfort, Pain**	Nothing	21(52.5%)	35 (83.3%)	56 (68.3%)	0.007
	A little	16 (40.0%)	5 (11.9%)	21 (25.6%)	
	A lot	3 (7.5%)	1 (2.4%)	4 (4.9%)	
	Very much	0 (0.0%)	1 (2.4%)	1(1.2%)	
**Embarrassed, Self-Conscious**	Nothing	2 (5.0%)	2 (4.8%)	4 (4.9%)	0.536
	A little	21 (52.5%)	26 (61.9%)	47 (57.3%)	
	A lot	10 (25.0%)	11 (26.2%)	21 (25.6%)	
	Very much	7 (17.5%)	3 (7.1%)	10 (12.2%)	
**Shopping/Housework Problems**	Nothing	8 (20.0%)	13 (31.0%)	21 (25.6%)	0.110
	A little	20 (50.0%)	25 (59.5%)	45 (54.9%)	
	A lot	10 (25.0%)	3 (7.1%)	13 (15.9%)	
	Very much	2 (5.0%)	1 (2.4%)	3 (3.7%)	
**Clothes**	Nothing	0 (0.0%)	0 (0.0%)	0 (0.0%)	0.867
	A little	5 (12.5%)	7 (16.7%)	12 (14.6%)	
	A lot	25 (62.5%)	25 (59.5%)	50 (61.0%)	
	Very much	10 (25.0%)	10 (23.8%)	20 (24.4%)	
**Social Activities**	Nothing	1 (2.5%)	0 (0.0%)	1 (1.2%)	0.243
	A little	20 (50.0%)	29 (69.0%)	49 (59.8%)	
	A lot	14 (35.0%)	10 (23.8%)	24 (29.3%)	
	Very much	5 (12.5%)	3 (7.1%)	8 (9.8%)	
**Sport**	Nothing	12 (30.0%)	12 (28.6%)	24 (29.3%)	0.006
	A little	11 (27.5%)	25 (59.5%)	36 (43.9%)	
	A lot	13 (32.5%)	4 (9.5%)	17 (20.7%)	
	Very much	4 (10.0%)	1 (2.4%)	5 (6.1%)	
**Work Study**	Nothing	10 (25.0%)	9 (21.4%)	19 (23.2%)	0.821
	A little	22 (55.0%)	27 (64.3%)	49 (59.8%)	
	A lot	6 (15.0%)	4 (9.5%)	10 (12.2%)	
	Very much	2 (5.0%)	2 (4.8%)	4 (4.9%)	
**Partner/Friends**	Nothing	14 (35.0%)	11 (26.2%)	25 (30.5%)	0.042
	A little	14 (35.0%)	25 (59.5%)	39 (47.6%)	
	A lot	9 (22.5%)	6 (14.3%)	15 (18.3%)	
	Very much	3 (7.5%)	0 (0.0%)	3 (3.7%)	
**Sexual Difficulties**	Nothing	22 (55.0%)	29 (69.0%)	51 (62.2%)	0.013
	A little	11 (27.5%)	13 (31.0%)	24 (29.3%)	
	A lot	5 (12.5%)	0 (0.0%)	5 (6.1%)	
	Very much	2 (5.0%)	0 (0.0%)	2 (2.4%)	
**Take Away Time**	Nothing	24 (60.0%)	22 (52.4%)	46 (56.1%)	0.025
	A little	9 (22.5%)	19 (45.2%)	28 (34.1%)	
	A lot	5 (12.5%)	1 (2.4%)	6 (7.3%)	
	Very much	2 (5.0%)	0 (0.0%)	2 (2.4%)	

**Table 4:** Differences between groups in the mean HDSS (Hyperhidrosis Disease Severity Scale) and DLQI (Dermatology Life Quality Index) scales in the reviews conducted.

	N	Mean/Standard Deviation	Significance
**Differenceat 1 month**			0.553
HDSS	40	2.1 ± 0.6	
100 U	42	1.9 ± 0.5	
DLQI 50 U	40	10.4 ± 5.9	
100 U	42	8.3 ± 2.9	
**Difference at 6 months**			0.066
HDSS 50 U	39	1.3 ± 0.9	
100 U	42	0.9 ± 0.7	
DLQI 50 U	39	6.3 ± 6.7	
100 U	42	3.8 ± 3.8	
**Difference at 9 months**			0.115
HDSS 50 U	28	1 ± 0.9	
100 U	24	0.7 ± 0.7	
DLQI 50 U	28	5.7 ± 6.2	
100 U	24	2.7 ± 3.7	
**Difference at >9 months**			0.085
HDSS 50 U	18	0.3 ± 0.6	
100 U	12	0 ± 0.4	
DLQI 50 U	18	1.8 ± 4.4	
100 U	12	0.2 ± 3.5	

**Table 5:** Results of Patient Global Impression of Improvement Scale, PGI-I, with doses of 50 U or 100 U of toxin per axilla.

	**50 U**	**100 U**	**Total**	**Significance**
**Very much better**				0.441
N	33	34	67	
%	82.5%	81.0%	81.7%	
**Much better**				0.441
N	6	8	14	
%	15.0%	19.0%	17.1%	
**A little better**				0.441
**N**	1	0	1	
**%**	2.5%	0.0%	1.2%	
**Total**				0.441
**N**	40	42	82	
**%**	100.0%	100.0%	100.0%	

## Data Availability

Data are available upon reasonable request.
